# Altered glutamatergic response and functional connectivity in treatment resistant schizophrenia: the effect of riluzole and therapeutic implications

**DOI:** 10.1007/s00213-019-5188-5

**Published:** 2019-02-28

**Authors:** Toby Pillinger, Maria Rogdaki, Robert A. McCutcheon, Pamela Hathway, Alice Egerton, Oliver D. Howes

**Affiliations:** 1 Institute of Psychiatry, Psychology and Neuroscience, King’s College London, London, England; 2 Medical Research Council London Institute of Medical Sciences, London, England; 3 Institute of Clinical Sciences, Faculty of Medicine, Imperial College London, London, England; 4 Department of Electrical and Electronic Engineering, Imperial College London, London, England

**Keywords:** Schizophrenia, Psychosis, Treatment resistant, Glutamate, Riluzole, Spectroscopy, MRS, Negative, Cognitive

## Abstract

**Rationale:**

Anterior cingulate cortex (ACC) glutamatergic abnormalities are reported in treatment-resistant schizophrenia (TRS) and implicated in functional dysconnectivity and psychopathology. Preclinical evidence indicates riluzole reduces synaptic glutamate. However, it is unknown whether riluzole can modulate glutamate metabolite levels and associated functional connectivity in TRS.

**Objectives:**

To examine the relationship between glutamatergic function and cortical connectivity and determine if riluzole can modulate glutamate metabolite levels and cortical functional connectivity in TRS.

**Methods:**

Nineteen TRS patients and 18 healthy volunteers (HV) underwent magnetic resonance imaging consisting of MR spectroscopy measuring ACC glutamate plus glutamine (Glx), fMRI measuring resting ACC-functional connectivity, and arterial spin labelling measuring regional cerebral blood flow (rCBF), and clinical measures. They then received 50 mg riluzole twice daily for 2 days when imaging was repeated.

**Results:**

Baseline (pre-riluzole) Glx levels were correlated directly with negative symptom severity (*r* = 0.49; *p* = 0.03) and inversely with verbal learning in TRS (*r* = − 0.63; *p* = 0.002), but not HV (*r* = − 0.24; *p* = 0.41). Connectivity between the ACC and anterior prefrontal cortex (aPFC) was correlated with verbal learning in TRS (*r* = 0.49; *p* = 0.04), but not HV (*r* = 0.28; *p* = 0.33). There was a significant group × time interaction effect on Glx levels (*p* < 0.05) and on ACC connectivity to the aPFC (*p* < 0.05, FWE-corrected). Riluzole decreased Glx and increased ACC-aPFC connectivity in TRS relative to HV. Change in Glx correlated inversely with change in ACC-aPFC connectivity in TRS (*r* = − 0.52; *p* = 0.02) but not HV (*r* = 0.01; *p* = 0.98). Riluzole did not alter rCBF (*p* > 0.05), indicating absence of a non-specific blood flow effect.

**Conclusion:**

Results indicate glutamatergic function and cortical connectivity are linked to symptoms and cognitive measures and that it is possible to pharmacologically modulate them in TRS.

**Electronic supplementary material:**

The online version of this article (10.1007/s00213-019-5188-5) contains supplementary material, which is available to authorized users.

## Introduction

Schizophrenia has a worldwide lifetime prevalence of approximately 1% (McGrath et al. 2008). It is a leading contributor to global disease burden, partly because many patients do not respond sufficiently to currently available treatments (Howes et al. 2017). Indeed, approximately two-thirds of patients with schizophrenia show a suboptimal symptomatic response to standard antipsychotic administration, which all target dopamine D2 receptors (Howes et al. 2017; Meltzer 1997). Future drug development therefore requires a greater understanding of the biological processes underlying the illness to identify new therapeutic targets (Howes and Kapur 2014).

Converging lines of evidence implicate glutamatergic dysfunction in the pathophysiology of schizophrenia (Javitt 2007; Ripke et al. 2014). The *N*-methyl-d-aspartate receptor (NMDAR) hypofunction model of schizophrenia proposes that dysfunction of NMDARs on parvalbumin-containing γ-aminobutyric acid–ergic interneurons results in disinhibition of excitatory pyramidal cells leading to an increase in glutamatergic activity (Lisman et al. 2008; Olney and Farber 1995; Stone et al. 2007). Administration of the NMDAR antagonist ketamine increases glutamatergic metabolites in the frontal cortex (Moghaddam et al. 1997; Stone et al. 2012); induces mental experiences characteristic of positive, negative, and cognitive symptoms of schizophrenia in healthy volunteers; and exacerbates psychotic symptoms in patients with schizophrenia (Cheng et al. 2018; Javitt and Zukin 1991). Meta-analysis of in vivo magnetic resonance spectroscopy (MRS) studies has shown an elevation in glutamate plus glutamine (Glx) across several brain regions in schizophrenia (Merritt et al. 2016), with some, although not all, studies observing that the magnitude of regional glutamate alterations correlates with the severity of negative and cognitive symptoms (Merritt et al. 2013).

There is emerging evidence that glutamate dysfunction may play a particular role in treatment-resistant symptoms (Egerton et al. 2017). Although not a universal finding (Goldstein et al. 2015), studies using proton magnetic resonance spectroscopy (^1^H-MRS) have observed that levels of glutamatergic metabolites are particularly elevated in the anterior cingulate cortex (ACC) in treatment-resistant schizophrenia (TRS) compared to levels in patients who respond to antipsychotic treatment, and healthy volunteers (Demjaha et al. 2014; Egerton et al. 2012; Goldstein et al. 2015; Mouchlianitis et al. 2016).

It has also been suggested that glutamatergic dysfunction could underlie cortical functional dysconnectivity in schizophrenia (Stephan et al. 2006). Indeed, schizophrenia is associated with decreased resting state functional connectivity (Dong et al. 2018), including deficits in functional connectivity between the ACC and frontal cortex that are present from onset of illness and the prodromal phase, and that are related to symptom severity (Lord et al. 2011). Moreover, reductions in ACC-prefrontal cortex functional connectivity have been observed in schizophrenia patients with persistent auditory hallucinations compared with patients without hallucinations, suggesting that alterations in ACC-prefrontal connectivity may contribute to at least some treatment-resistant symptoms (Alonso-Solis et al. 2015).

Riluzole (2-amino-6-trifluormethoxy benzothiazole) is a drug licenced for amyotrophic lateral sclerosis that acts to reduce synaptic release of glutamate by inhibiting voltage-gated sodium channels and calcium currents (Bellingham 2011; Doble 1996). It also enhances astrocytic glutamate re-uptake (Frizzo et al. 2004), increases cortical glutamate metabolism (Chowdhury et al. 2008), and reduces the amount of releasable presynaptic glutamate (Lazarevic et al. 2018). Riluzole thus represents a promising agent to target glutamatergic dysfunction in schizophrenia. Indeed, a recent randomised controlled trial in 50 patients with schizophrenia and treatment-resistant symptoms observed that adjunctive riluzole significantly decreased negative symptom severity within 4 weeks compared to a placebo group (Farokhnia et al. 2014). The mechanism underlying this effect is unknown, but riluzole has been shown to alter ACC glutamatergic metabolite concentrations in autism spectrum disorder, increasing prefrontal concentrations of Glx relative to gamma-aminobutyric acid (GABA) (Ajram et al. 2017), and in bipolar depression, increasing the ACC glutamine to glutamate ratio (Brennan et al. 2010). Moreover, in ASD, riluzole reduces abnormal prefrontal connectivity (Ajram et al. 2017). However, it is unknown if riluzole is able to alter glutamatergic signalling or cortical connectivity in schizophrenia.

In view of this, we aimed to test the hypotheses that riluzole would reduce Glx levels and increase cortical connectivity in individuals with schizophrenia and antipsychotic-resistant symptoms, and that these effects would be related to each other. We used a healthy volunteer group to enable normative comparisons and control for non-specific effects. Based on preclinical evidence that riluzole does not alter glutamatergic indices when glutamate function is normal (Rizzo et al. 2017), we predicted that riluzole would have no effects on glutamatergic metabolites or ACC connectivity in healthy volunteers.

## Methods

### Participants and clinical measures

Twenty-one participants meeting DSM-IV criteria for schizophrenia were recruited from outpatient services within the South London and the Maudsley NHS Foundation Trust (Beck et al. 2014). Nineteen healthy volunteers with no history of psychiatric illness were recruited from the local population to provide a normative comparison. Exclusion criteria for all participants were as follows: inability to provide written informed consent; co-morbid drug or alcohol abuse/dependence; a history of liver disease or transaminitis > 2 times the upper limit of normal (owing to the potential for riluzole to cause liver dysfunction (Castells et al. 1998)); any contraindication to MRI scanning at 3 T (e.g. metallic implants); any comorbidity that could compromise scanning safety (e.g. severe asthma); pregnancy/breast feeding; and the use of medication with recognised effect on glutamatergic signalling, including clozapine, lamotrigine, lithium, carbamazepine, opiates, and psychostimulants.

Treatment-resistant schizophrenia was defined as presence of at least one positive and one negative symptom rated as ≥ 4 on the Positive and Negative Syndrome Scale (PANSS) (Kay et al. 1987), indicative of at least moderate severity, and a score of < 60 on the Global Assessment of Functioning scale (GAF) (APA 2013) indicative of at least moderate functional impairment, despite 2 trials of an antipsychotic. To provide insight into the range of illness severity within the TRS cohort, severity of illness was defined according to criteria set out by Leucht and colleagues that classifies total PANSS scores of 58–74 as mild-moderate illness, 75–94 as moderate-marked illness, and 95–115 as marked-severe illness (Leucht et al. 2005). A sufficient antipsychotic trial was defined as one given for at least 6 weeks with evidence of concordance (based on examination of patient records) and at a target dose recommended by the relevant manufacturer’s summary of product characteristics/at a total daily dose equivalent to or greater than 600 mg chlorpromazine. Patients were required to be on a stable antipsychotic regimen, with no change in treatment dose in the 6 weeks prior to study participation. Antipsychotic plasma levels were measured to assess concordance, as previously described (McCutcheon et al. 2018). This approach to defining treatment-resistant schizophrenia conformed with at least the minimum requirements provided by Treatment Response and Resistance in Psychosis (TRRIP) working group consensus guidelines (Howes et al. 2017), summarised in eTable 1. Clinical Global Impression (CGI)-Severity (Guy 1976) scores were also recorded. All participants underwent neurocognitive testing with the Rey Auditory and Verbal Learning Test (AVLT) (Schmidt 1996), a well-established tool to assess cognitive functioning in schizophrenia (Zaytseva et al. 2018). AVLT total score (the number of words correctly recalled, summed across the five immediate recall trials) was used to assess verbal-learning performance (Karilampi et al. 2007), a recognised neurocognitive deficit in TRS (Joober et al. 2002). Participants underwent two MRI scans. On both scan days, all participants underwent urine testing for cocaine, amphetamine, cannabis, opiate, and benzodiazepine use.

### Administration of riluzole

We used a 2-day riluzole challenge because a previous ^1^H-MRS study showed an effect of riluzole on ACC glutamate and glutamine levels in bipolar depression using this treatment duration (Brennan et al. 2010). After the baseline MRI scan, the 2-day course of riluzole was given at a dose of 50 mg every 12 h, the dose and frequency recommended in the treatment of ALS (Miller et al. 2012). Since peak plasma levels of riluzole occur 1–1.5 h after oral administration (LeLiboux et al. 1997), the final (fourth) dose was taken 1.5 h before the second scan commenced. Adherence was ensured by SMS messaging reminders to participants, and inspection of medication containers at presentation to the follow-up scan.

### ^1^H-MRS acquisition

**Fig. 1:**
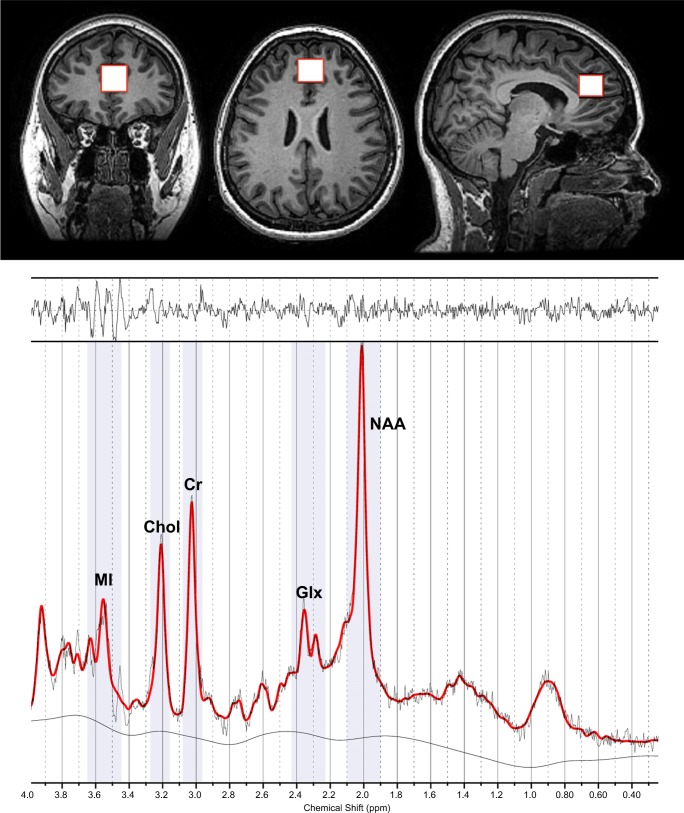
^1^H-MRS voxel position and example spectra in the anterior cingulate cortex. NAA, *N*-acetylaspartate; Glx, glutamate + glutamine; Cr, creatine; Chol, choline; MI, myoinositol

Scans were acquired using MRI at 3 T (General Electric, Chicago, IL, USA). All scans were performed at the same time of day (mid-morning). Each scanning session commenced with a localizer, standard axial T2-weighted fast spin echo scan (TR/TE = 4380/55.72) and a T1-weighted structural scan (TR/TE = 7.312/3.01). The T1-weighted image was used to plan ^1^H-MRS voxel placement, and for calculation of ^1^H-MRS voxel tissue content. The ^1^H-MRS voxel was placed in the anterior cingulate cortex (ACC). The ACC voxel was defined from the midline sagittal localizer, with the centre of the 20 mm × 20 mm × 20 mm voxel placed 16 mm above the genu of corpus callosum perpendicular to the AC–PC line (Fig. 1). ^1^H-MRS spectra (Point RESolved Spectroscopy; TE = 30 ms; TR = 3000 ms; 96 averages; bandwidth = 5 kHz, number of data points = 4096) were acquired using the standard GE PROBE (proton brain examination) sequence. Additional unsuppressed water reference spectra (16 averages) were acquired for eddy current correction and water scaling.

### pCASL acquisition

To determine if changes in ACC-Glx or connectivity were secondary to changes in regional blood flow, we assessed the effect of riluzole on cerebral blood flow in TRS patients and HV using arterial spin labelling MR imaging. For measurement of regional cerebral blood flow (rCBF), a 3D pseudo-continuous ASL (pCASL) acquisition was used. Arterial blood was labelled using a long (1.5 s) train of adiabatic radio frequency pulses. After a post-labelling delay of 1.5 s, perfusion images were acquired with a 3D Fast Spin Echo (FSE) stack-of-spirals multi-shot readout (TE/TR = 32 ms/5500 ms; ETL = 64) (Dai et al. 2008). CBF maps were computed in physiological units of ml blood per 100 mg of tissue per minute, with a voxel size of 1 × 1 × 3 mm^3^. During the scan, participants were instructed to keep their eyes open and look at a fixation cross.

### Resting state fMRI acquisition

Resting state fMRI was acquired using a multi-echo echo-planar imaging (ME-EPI) sequence: TR = 2.5 s; TE = 12, 28, 44, 70 ms; 240 time-points; slice thickness = 3 mm; slice spacing = 4 mm; spatial positions, 32; flip angle 80°; field of view 240 mm; matrix size 64 × 64; scan time 10 min. During the scan, participants were instructed to keep their eyes open and look at a fixation cross.

### ^1^H-MRS analysis

Spectra were analysed using LC Model version 6.3-1L 44. Voxel grey matter (GM), white matter (WM), and cerebrospinal fluid (CSF) content for each subject were derived by extracting the location of the voxel from the spectra file headers and using an in-house program to calculate the percentage of GM, WM, and CSF using the segmented T1-weighted images. Segmentation was performed using the ‘segment’ function of SPM12. Water-scaled metabolites were corrected for CSF using the formula: metabolite corrected = metabolite concentration × [proportion WM + (1.21 × proportion GM) + (1.548 × proportion CSF)]/(proportion WM + proportion GM). The formula assumes a CSF water concentration of 55,556 mol/m^3^ with the LCModel default brain water concentration of 35,880 mol/m^3^ (Gasparovic et al. 2006; Kreis et al. 1993). Poor-quality scans, as defined by poorly fitted metabolite peaks (Cramér–Rao minimum variance bounds > 20%, and signal to noise ratio < 8, as reported by LCModel) were excluded from further analysis.

### pCASL analysis

Computation of the CBF values was performed in the scanner following the methodology outlined in the recent ASL consensus paper (Alsop et al. 2015). Individual CBF maps were transformed to Montreal Neurological Institute (MNI) space using the Automatic Software for ASL Processing (ASAP) toolbox (Abad et al. 2016) running in SPM-8 under Matlab 6.5. Default pre-processing options were used for skull-stripping, co-registration to the subject’s 3D anatomical scan, and normalisation to the MNI template based on unified segmentation. The normalised maps were finally smoothed using an 8-mm kernel. Segmentation was performed using the ‘segment’ function of SPM12.

### Resting state fMRI analysis

After realignment and slice timing correction, multi-echo independent component analysis was used to denoise the resting state data (Kundu et al. 2013). After performing an independent component analysis on the unprocessed resting fMRI data, the dependence of each component on echo time (TE) is quantified. Genuine BOLD T2* signal is linearly related to TE, whereas artefactual signal is not. As a result, it is then possible to separate resting state networks from noise components. The time courses from the non-BOLD components are then used as regressors for data cleaning, along with white matter and CSF time courses. Temporal band bass filtering was performed using FSL with sigma = 50 (Smith et al. 2004). Normalisation to MNI space was then performed using the CONN v18 functional connectivity toolbox (Whitfield-Gabrieli and Nieto-Castanon 2012). Segmentation was performed using FSL FAST.

### Statistical analysis

The effects of riluzole on ^1^H-MRS metabolite levels in TRS compared with HV over time in the ACC were determined using a two-way (group × time) repeated measures ANOVA, with the primary outcome defined a priori as Glx levels. Outliers in each group (patient and control, pre- and post-riluzole) were identified using the Tukey method (Tukey 1977), and analyses performed with these removed. Where a significant group × time interaction was recorded, post hoc unpaired *t* tests were performed to examine differences in Glx between groups pre- and post-riluzole, and paired *t* tests performed to examine differences in Glx within groups over time. Exploratory analyses were also performed for changes in glutamate and *N*-acetylcysteine levels over time (both CSF-corrected and referenced to creatine).

The effects of riluzole on rCBF in TRS compared with HV were also examined using a repeated measures ANOVA implemented in SPM-8. We performed SPM analyses both at a whole brain level (cluster defining threshold *p* < 0.001, uncorrected for multiple comparisons), and in the ACC. The ACC region of interest was created of the same dimension as the MRS voxel (Fig. 1). We employed the uncorrected threshold so as to increase the sensitivity to potential effects of riluzole upon blood flow.

For the resting state connectivity analysis, voxel-wise connectivity maps for each participant were derived by computing Pearson correlations between the signal average over each seed region, and the signal at each voxel over the entire brain. These were then converted to normally distributed Fisher’s *z* maps to allow second-level general linear model analyses. At the second level, a seed to voxel analysis was performed with a view to examining the effect of riluzole on ACC-frontal connectivity. Six ACC seeds were selected a priori from 32 ACC seeds previously characterised by Margulies and colleagues (Margulies et al. 2007), who in observing functional heterogeneity within the ACC identified six seeds with evidence of functional connectivity to the frontal cortex (see eAppendix 1 and eTable 2 for further details). Connectivity maps between groups (TRS group and HV group, pre- and post-riluzole) were contrasted with each other for the six ACC seeds. A cluster was considered statistically significant if it passed a cluster defining threshold of *p* < 0.001 and cluster-level threshold of *p* < 0.05 FWE corrected.

Spearman’s correlation coefficients were used to examine the relationship between changes in imaging variables over time (e.g. changes in ACC ^1^H-MRS metabolite levels and changes in ACC-cluster connectivity). Moreover, to help interpret the clinical relevance of our findings, pre-riluzole, Spearman’s correlation coefficients were also used to examine the relationship between imaging variables (e.g. ACC ^1^H-MRS metabolite levels) and clinical (PANSS) and neurocognitive (AVLT) scores. Spearman correlation coefficients were employed owing to the measure being robust to the influence of outliers (King 1992). All non-SPM statistical analysis was performed using SPSS software (version 22.0, Chicago, IL), for which statistical significance was defined as *p* < 0.05.

## Results

### Sample characteristics

Participant demographic and clinical measures are presented in Table 1. Riluzole was well tolerated in all participants and no adverse effects were reported. Of the patients with TRS, nine were receiving long-acting injectable antipsychotic medication. Five patients were receiving risperidone, one zuclopenthixol decanoate, two aripiprazole, five paliperidone, five olanzapine, three amisulpride, and one quetiapine. Two patients were receiving dual antipsychotic treatment. Plasma antipsychotic levels were in the therapeutic range for all participants. On both scan days, all participants tested negative on urine testing for cocaine, amphetamine, cannabis, opiates, and benzodiazepine use.

**Table 1:** Demographics and where appropriate clinical measures of patients and healthy volunteers included in the primary repeated measures ^1^H-MRS analysis. Where appropriate, data are reported as mean (standard deviation). *CGI* Clinical Global Impression score, *GAF* Global Assessment of Functioning score, *PANSS* Positive and Negative Syndrome Scale scores, *AVLT* Auditory and Verbal Learning Test

	Patients (*n* = 19)	Healthy volunteers (*n* = 18)	Statistic
Age (years)	39.68 (10.92)	36.28 (9.17)	*t* = 1.02; df = 35; *p* = 0.31
Gender (female/male)	3/16	3/15	*χ*^2^ = 0.01; *p* = 0.94
AVLT score	35.58 (15.36)	59.86 (11.69)*	*t* = 4.95; df = 31; *p* < 0.0001
PANSS—positive	18.47 (3.03)		
PANSS—negative	19.58 (5.48)		
PANSS—general	34.42 (7.96)		
PANSS—total	72.47 (10.19)		
CGI	4.15 (0.77)		
GAF	52.1 (9.98)		
Severity of illness	Mild-moderate, 7/19 (37%) Moderate-marked, 11/19 (58%) Marked-severe, 1/19 (5%)		

*Data available for 14 healthy volunteers

Pre- and post-riluzole MRI datasets were available in 19 of the 21 patients, as 2 participants chose not to continue with the study after the first scan. Pre-and post-riluzole MRI datasets were available in 18 of the 19 healthy controls, as scanner failure precluded a follow-up scan for one participant. Nineteen TRS patients and 18 healthy volunteers were included in 1H-MRS analyses. Nineteen TRS patients and 18 healthy volunteers were included in rCBF analyses, and 19 TRS patients and 17 healthy volunteers were included in resting state fMRI analyses (owing to scanner failure with one resting state sequence in the HV group). For those TRS patients who completed the study, 37% patients presented with mild-moderate symptoms, 58% presented with moderate-severe symptoms, and 5% with marked-severe symptoms. There were no significant differences in values relating to ^1^H-MRS data quality or voxel tissue content in patients compared with controls over time (eTable 3). For all ^1^H-MRS data, data were reported for which all individual Cramér Rao Lower Bounds were below 20%, signal to noise ratio values were above 8, and no spectra were excluded based on poor quality.

### Effect of riluzole on glutamate metabolites, ACC-frontal connectivity, and rCBF

**Fig. 2:**
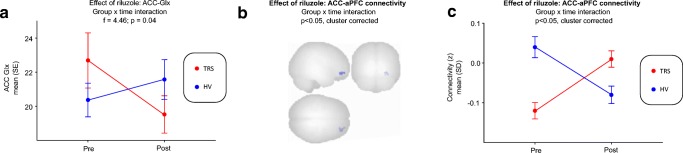
Effect of riluzole on voxel glutamate metabolite levels and anterior cingulate cortex (ACC) connectivity in patients with treatment-resistant schizophrenia (TRS) compared with healthy volunteers (HV). **a** There was a significant group by time interaction for Glx (glutamate and glutamine) levels in the ACC with administration of riluzole (*p* = 0.04). **b** There was a significant group × time interaction for connectivity between the ACC seed and a cluster within the anterior prefrontal cortex (aPFC, *p* < 0.05, cluster corrected). **c** Change in ACC-functional connectivity *z* scores in TRS compared with HV pre- and post-riluzole for the aPFC cluster represented in **b** (*p* < 0.05, cluster corrected)

There was a significant group by time interaction for Glx levels in the ACC (*f* = 4.46; *p* = 0.04; Fig. 2a; Table 2). Outliers identified using the Tukey method are demonstrated in eFigure 1. Results were similar after removal of outliers (*f* = 7.16; *p* = 0.01). On post hoc analysis, ACC-Glx levels pre- and post-riluzole were not significantly different in TRS compared with HV, nor was there any difference in ACC-Glx within groups over time (all *p* ≥ 0.05, eTable 4). Specifically, riluzole was associated with a numerical decrease in ACC-Glx in TRS, although statistical significance was not reached (*t* = − 2.08; *p* = 0.05). There were no significant group by time effects for ACC glutamate or N-acetyl asparate levels (eTable 5). There was no significant group by time interaction for creatine-scaled Glx levels (*f* = 3.27; *p* = 0.08), nor for creatine-scaled glutamate or *N*-acetyl aspartate levels (eTable 6). There were no significant group by time effects for rCBF, either in the ACC or at whole brain level (Table 2). For ACC and whole brain CBF, there was no significant difference between TRS and HV pre- and post-riluzole, nor was there any difference in CBF within groups over time (all *p* > 0.05, eTables 7 and 8).

**Table 2:** Cerebrospinal fluid (CSF) corrected glutamate + glutamine (Glx) values in the anterior cingulate cortex (ACC), and cerebral blood flow in the anterior cingulate cortex and whole brain pre- and post-riluzole. Data are presented as mean (standard deviation) and the statistical analysis shows the results of the group by time interaction tested using a repeated measures ANOVA

	Patients		Healthy volunteers		
	Pre	Post	Pre	Post	Group × time interaction
ACC Glx	22.69 (7.05)	19.52 (4.79)	20.37 (4.19)	21.57 (4.97)	*F* = 4.46; df = 35; *p* = 0.04
ACC CBF	44.99 (7.51)	45.13 (7.34)	44.03 (6.36)	43.93 (5.02)	*F* = 0.02; df = 35; *p* = 0.90
Whole brain CBF	40.17 (6.67)	40.85 (7.22)	39.48 (5.81)	39.52 (5.25)	*F* = 0.15; df = 35; *p* = 0.70

There was a significant group × time interaction for connectivity between the ACC seed sited at MNI coordinate (± 5, 27, 21) with a cluster sited within the right anterior prefrontal cortex (aPFC) (Fig. 2b). For this cluster, at baseline, ACC-frontal connectivity was lower in patients compared with healthy volunteers, and this outcome reversed following riluzole (Fig. 2c). We did not observe a significant interaction for the five other ACC seeds examined.

### Correlations between Glx and functional connectivity

**Fig. 3:**
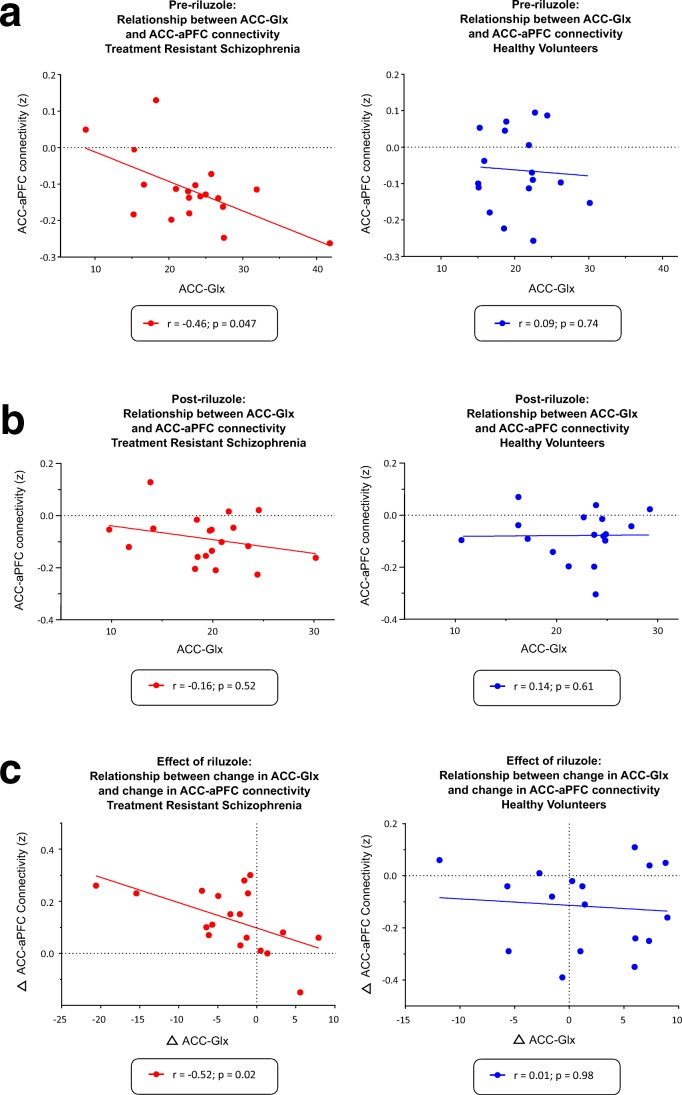
Association between changes in anterior cingulate cortex (ACC) glutamate and glutamine (Glx) levels and changes in ACC-functional connectivity (with anterior prefrontal cortex (aPFC) cluster defined in Fig. 2b) following riluzole. **a** Baseline ACC-Glx correlates inversely with degree of functional connectivity between ACC and aPFC cluster in TRS (*r* = − 0.46; *p* = 0.047) but not HV (*r* = 0.09; *p* = 0.74). **b** Following riluzole challenge, no significant association is observed between ACC-Glx and functional connectivity between ACC and aPFC cluster in TRS (*r* = − 0.16; *p* = 0.52) or HV (*r* = 0.14; *p* = 0.61). **c** Change in ACC-Glx levels correlated inversely with change in functional connectivity between the ACC and aPFC cluster in the TRS group (*r* = − 0.52; *p* = 0.02) but not HV group (*r* = 0.01; *p* = 0.98). Lines represent best fit regression and *r* values represent Spearman rank correlation coefficients

Pre-riluzole, ACC-Glx correlated inversely with degree of ACC-aPFC functional connectivity in TRS (*r* = − 0.46; *p* = 0.047), but not HV (*r* = 0.09; *p* = 0.74) (Fig. 3a). Post-riluzole, no significant association was observed between ACC-Glx and ACC-aPFC functional connectivity in TRS (*r* = − 0.16; *p* = 0.52) or HV (*r* = 0.14; *p* = 0.61) (Fig. 3b). Changes with riluzole in functional connectivity between the ACC and aPFC cluster were inversely correlated with changes in ACC-Glx levels in TRS patients (*r* = − 0.52; *p* = 0.02), but not HV (*r* = 0.01; *p* = 0.98) (Fig. 3c).

### Correlations between imaging variables and symptom severity

**Fig. 4:**
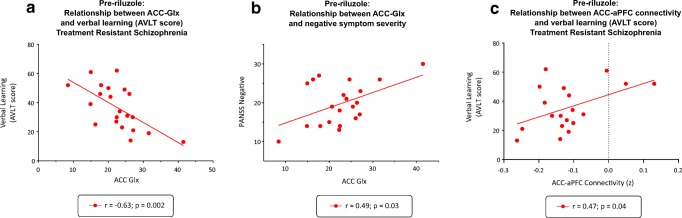
Pre-riluzole associations between anterior cingulate cortex (ACC) glutamate and glutamine (Glx) levels/functional connectivity *z* scores (between ACC and aPFC cluster identified in Fig. 2b) with Auditory Verbal Learning Test (AVLT) scores and negative symptom severity. **a** Higher levels of ACC Glx are associated with reduced AVLT scores in TRS (*r* = − 0.63; *p* = 0.002). **b** In TRS, higher levels of ACC Glx are associated with greater severity of negative symptoms (*r* = 0.49; *p* = 0.03). **c** Increased ACC-functional connectivity is associated with higher AVLT scores in TRS (*r* = 0.49; *p* = 0.04). Lines represent best-fit regression and *r* values represent Spearman rank correlation coefficients

Pre-riluzole, increased ACC-Glx was associated with lower verbal learning scores in TRS (*r* = − 0.63; *p* = 0.002) but not HV (*r* = − 0.24; *p* = 0.41) (Fig. 4a). Moreover, increased ACC-Glx was associated with elevated PANSS negative scores in TRS (*r* = 0.49; *p* = 0.03) (Fig. 4b). Pre-riluzole, lower ACC-aPFC functional connectivity was associated with lower verbal learning scores in TRS (*r* = 0.47; *p* = 0.04), but not HV (*r* = 0.28; *p* = 0.33) (Fig. 4c). There were no other significant correlations between baseline PANSS (positive, general, or total) or AVLT scores and ACC-Glx/ACC-aPFC connectivity (all *p* > 0.05, eTable 9).

## Discussion

Our main findings are that riluzole reduces ACC-Glx concentrations and normalises ACC-frontal hypoconnectivity in patients with TRS relative to healthy volunteers. Moreover, changes in ACC-Glx concentrations were associated with changes in ACC-frontal connectivity in TRS. At baseline, greater ACC-Glx concentrations were associated with more severe negative symptoms and poorer cognitive performance, adding to prior evidence for magnitude of regional glutamate alterations in schizophrenia correlating with severity of negative and cognitive symptoms (Merritt et al. 2013). We did not observe any difference in rCBF in the ACC or whole brain of patients or controls in response to riluzole, suggesting that observed changes in Glx and functional connectivity are not secondary to non-specific changes in cerebral blood flow.

### Implications for understanding and treating schizophrenia

A previous clinical study in patients with schizophrenia and treatment-resistant symptoms showed that riluzole (at the same daily dose used in the current study) added to risperidone resulted in a significant improvement in negative symptoms after 4 week-treatment relative to placebo (Farokhnia et al. 2014). Our findings extend this study by showing for the first time to our knowledge that riluzole has schizophrenia-specific effects on glutamatergic signalling and brain connectivity. Taken with our finding that negative and cognitive symptoms were directly correlated with brain glutamate function, this indicates riluzole is acting to target dysfunctional brain systems in schizophrenia linked to negative and cognitive symptoms, supporting its further evaluation as an adjunctive treatment for schizophrenia.

The reduction in Glx levels following riluzole in TRS relative to HV may suggest illness-related differences in glutamatergic signalling that become apparent when challenged with riluzole. The molecular targets of riluzole may include presynaptic calcium channels (Doble 1996) and/or excitatory amino acid transporters (EAAT) (Frizzo et al. 2004), and interaction at either or both of these sites could potentially impact on the ^1^H-MRS Glx signal. A preclinical study using ^13^C-MRS found that riluzole administration enhanced prefrontal cortex glutamate metabolism, suggesting increased glutamate release and cycling through the glutamate/GABA/glutamine pathway (Chowdhury et al. 2008). This is counterintuitive since riluzole reduces presynaptic release of glutamate (Bellingham 2011; Doble 1996). However, since riluzole can also increase glutamatergic clearance from the extra-synaptic space (Frizzo et al. 2004), the net effect at the level of an MRS voxel, and after 2 days of riluzole administration, may still be an overall reduction in Glx. A limitation of our ^1^H-MRS methodology is that we are unable to measure glutamine and cannot examine glutamate cycling, as would be possible with ^13^C-MRS. A recent ^13^C-MRS human study examining the acute effects of ketamine found increased prefrontal glutamate release (a ‘glutamate surge’), and loss of neurotransmission fidelity (i.e. a mismatch between pre-synaptic glutamate release and post-synaptic activity) which was associated with the induction of psychotomimetic experiences (Abdallah et al. 2018). If riluzole does indeed ameliorate synaptic NMDA receptor-mediated neurotransmission in disease states characterised by hyperglutamatergia, then our observation of normalisation of ACC-aPFC connectivity in TRS with riluzole may reflect improvements in fidelity of neurotransmission.

Previous drug studies targeting glutamatergic neurotransmission in schizophrenia have been disappointing. Broadly, two families of drugs have been examined: drugs that increase NMDA receptor activity (such as glycine and bitopertin) (Buchanan et al. 2007; Singer et al. 2015) and drugs that inhibit glutamate release (mGluR2/3 agonists) (Li et al. 2015). Riluzole’s mechanism of action is distinct from those previously trialled, and based on the neurochemical and neurofunctional outcomes of the present study may represent a viable novel therapeutic avenue, especially in treatment-resistant schizophrenia.

The effect of riluzole on connectivity between the ACC and frontal cortex was significant for one seed in the ACC. This may reflect the recognised functional heterogeneity of the ACC (Margulies et al. 2007). The ACC seed identified in this study has previously been observed to show functional connectivity with prefrontal regions associated with higher order cognitive functions (e.g. working memory) (Margulies et al. 2007), which complements our observation of a pre-riluzole direct relationship between degree of ACC-aPFC connectivity and magnitude of verbal learning scores in patients. However, our study was not designed to test the specificity of sub-regional effects, and further work mapping the functional connectivity of the ACC in TRS is required to determine this. Although baseline Glx levels were numerically higher in the schizophrenia group than the healthy volunteers, consistent with some prior evidence (Merritt et al. 2016), this was not statistically significant (*p* = 0.24), which could be due to a lack of power. Indeed, the effect size for baseline ACC-Glx difference between patients and healthy volunteers was 0.40. For two independent samples, an effect size of 0.40 requires a total sample size of 200 to provide 80% power to detect a significant difference between groups (*α* = 0.05, two tailed).

### Strengths and limitations

The strength of this study is that we assured treatment adherence (eTable 1) and excluded psychoactive substance use during the study, which reduces the heterogeneity of the sampled patient population (Howes and Kapur 2014). Furthermore, the use of multimodal imaging techniques provides comprehensive insight into neurochemical and neurofunctional alterations associated with riluzole administration.

The absence of a placebo condition means results cannot be attributed to riluzole specifically. However, the inclusion of a healthy control group controls for non-specific effects on imaging outcomes, and the fact that subjects were blind to the hypotheses and the study used imaging outcomes makes it unlikely that outcomes were confounded by placebo effects. Nevertheless, it would be useful to test this further with the inclusion of a placebo group. Although psychopathology scores were assessed pre-riluzole, repeat measures were not recorded post-riluzole. This was based on evidence from the only previous study to examine the clinical efficacy of riluzole in schizophrenia which did not observe significant improvements in psychopathology until 4 weeks of treatment (Farokhnia et al. 2014). Future longitudinal studies should therefore include re-assessments of psychopathology, alongside neuroimaging.

Although participants were excluded if receiving medication with recognised glutamatergic activity, there is evidence that non-clozapine antipsychotic administration can reduce ACC-glutamate metabolite levels (Egerton et al. 2017; Egerton et al. 2018). While it is not generally feasible to recruit a cohort of individuals with TRS who are medication free, the fact that this is a repeated measures study and there were no changes in antipsychotic treatment during the study suggests the changes in Glx observed with riluzole are unlikely to be related to medication effects. As we did not include a treatment-responsive patient group for comparison, we cannot specifically attribute our findings to treatment-resistant schizophrenia. Further work is required to determine whether the effects of riluzole on Glx and ACC-frontal connectivity may differ in patients who respond well to antipsychotic medication compared to those with treatment-resistant illness.

Although the observed group × time interaction for ACC-Glx was significant, the effect of riluzole in decreasing ACC-Glx in TRS in absolute terms was at a trend significance level (*t* = 2.08; *p* = 0.05). This may reflect insufficient power in the current study. Moreover, results of correlation analyses with relatively small sample sizes should be interpreted with caution, and replication of our findings in larger cohorts is required.

A limitation of ^1^H-MRS at 3-T field strength is the inability to reliably quantify glutamine concentrations, owing to overlapping resonances between glutamate and glutamine. Thus, it was not possible to examine the relative changes in glutamine and glutamate concentrations in response to riluzole as has previously been performed (Brennan et al. 2010). Furthermore, we are unable to comment on the relative contribution of glutamate or glutamine to the ACC changes observed. Finally, as a methodology, ^1^H-MRS is limited by an inability to precisely identify the location of glutamatergic metabolites within a region of interest (i.e. intracellular versus extracellular, presynaptic versus postsynaptic, and neuron versus astrocyte/glia).

## Conclusion

Our findings add to evidence that glutamatergic dysfunction contributes to the pathophysiology of schizophrenia by showing that negative and cognitive symptoms are directly associated with levels of Glx, and that a glutamatergic modulator, riluzole, modulates Glx and frontal cortical connectivity in patients relative to controls. Future studies incorporating neuroimaging are required to investigate if riluzole-associated alterations in frontal glutamatergic metabolites and associated functional connectivity persist over longer treatment periods and are associated with clinical efficacy to help inform the potential of riluzole as an adjunctive treatment for schizophrenia.

### Funding/support

This study was funded by grants MC-A656-5QD30 from the Medical Research Council-UK, 666 from the Maudsley Charity 094849/Z/10/Z from the Brain and Behavior Research Foundation, and Wellcome Trust (Dr Howes) and the National Institute for Health Research Biomedical Research Centre at South London and Maudsley National Health Service Foundation Trust and King’s College London. R.M.’s work is supported by the Wellcome Trust (no. 200102/Z/15/Z).

## Electronic supplementary material

ESM 1 (DOCX 96 kb)
