# Supplementary material for: Altered glutamatergic response and functional connectivity in treatment resistant schizophrenia: the effect of riluzole and therapeutic implications
Source: Psychopharmacology (Berl). 2019 Feb 28;236(7):1985–97. doi: 10.1007/s00213-019-5188-5 (PMC6642056; doi:10.1007/s00213-019-5188-5)
Supplement: Supplementary file 1 — (DOCX 96 kb) [file 213_2019_5188_MOESM1_ESM.docx]

**SUPPLEMENTARY INFORMATION**

**CONTENTS**

**Page 2** eTable 1: Assessment of appropriate definition of treatment resistant schizophrenia employed

eAppendix 1: Description of seeds used in functional connectivity analysis

eTable 2: Seed regions used in anterior cingulate cortex connectivity analyses.

**Page 3** eTable 3: Signal-to-noise ratio, Cramér Rao Lower Bounds, and voxel white matter, grey matter, and CSF fractions in the anterior cingulate cortex pre- and post- riluzole

eTable 4: Post-hoc pre- vs post-riluzole comparison of anterior cingulate cortex Glutamate + glutamine (Glx) levels in patients and controls.

eTable 5: Exploratory cerebrospinal fluid corrected metabolite values (glutamate and N-acetyl aspartate) in the anterior cingulate cortex pre- and post-riluzole.

eTable 6: Creatine scaled metabolite values in the anterior cingulate cortex before and after riluzole.

**Page 4** eTable 7: Post-hoc examination of effect of riluzole on whole brain cerebral blood flow

eTable 8: Post-hoc examination of effect of riluzole on anterior cingulate cortex cerebral blood flow

**Page 5** eTable 9: Relationship between baseline (pre-riluzole) ACC-Glx levels and clinical/cognitive rating scales, and ACC-BA10 functional connectivity and clinical/cognitive rating scales

eFigure 1: Outlier identification for ^1^H-MRS analysis

Supplementary References

| Domain and Subdomain | Minimum Requirement | Optimum Requirement | Achieved in current study? |
| --- | --- | --- | --- |
| Current symptoms: Assessment | Interview using standardized rating scale (e.g., PANSS, BPRS, SANS, SAPS) | Prospective evaluation of treatment using a standardized rating scale | Minimum requirement |
| Current symptoms: Severity | At least moderate severity | At least moderate severity and 20% symptom reduction during a prospective trial or observation of ≥6 weeks | Minimum requirement |
| Current symptoms: Duration | ≥12 weeks | ≥12 weeks; specify duration of treatment resistance | Minimum requirement |
| Current symptoms: Functioning | At least moderate functional impairment measured using a validated scale | At least moderate functional impairment, measured using a validated scale (e.g., SOFAS) | Optimum requirement |
| Adequate treatment Assessment of past response | Information to be gathered from patient/carer reports, staff and case notes, pill counts, and dispensing charts | Same as for minimum requirement | Optimum requirement |
| Duration of symptoms | ≥6 weeks at a therapeutic dosage | Same as for minimum requirement | Optimum requirement |
| Antipsychotic Dosage | Equivalent to $600 mg of chlorpromazine per day. | Same as for minimum requirement | Optimum requirement |
| Current antipsychotic adherence | Adherence should be assessed using at least two sources (pill counts, dispensing chart reviews, and patient/carer report). Antipsychotic plasma levels monitored on at least one occasion. | Same as the minimum criteria, with the addition of trough antipsychotic serum levels measured on at least two occasions separated by at least 2 weeks (without prior notification of patient) | Minimum requirement |
| Symptom domain | Positive, negative, cognitive | Same as for minimum requirement | Optimum requirement |

**eTable 1:** Assessment of appropriate definition of treatment resistant schizophrenia used in this study, as defined by Treatment Response and Resistance in Psychosis (TRRIP) working group consensus guidelines.^1^

**eAppendix 1**

**Connectivity analysis**

Given the role of the frontal cortex in executive functioning, we chose the 6 ACC seeds which had previously been demonstrated to functionally connect to this region.^2^ Seeds were of 3.5 mm diameter, and bilateral pairs were examined together in the analysis.

| Seed | x | y | z |
| --- | --- | --- | --- |
| 1 | +5/-5 | +10 | +33 |
| 2 | +5/-5 | +19 | +28 |
| 3 | +5/-5 | +27 | +21 |
| 4 | +5/-5 | +14 | +42 |
| 5 | +5/-5 | +25 | +36 |
| 6 | +5/-5 | +34 | +28 |

**eTable 2:** Seed regions used in anterior cingulate cortex connectivity analyses.

| Spectral Quality | | | | | |
| --- | --- | --- | --- | --- | --- |
|  | **Patients** | | **Healthy volunteers** | |  |
|  | **Pre** | **Post** | **Pre** | **Post** | **Group x Time Interaction** |
| Signal to noise ratio | 22.89 (5.34) | 22.00 (4.15) | 23.33 (6.29) | 24.39 (4.85) | **F = 1.54, df = 35; p = 0.22** |
| White matter | 0.12 (0.10) | 0.12 (0.05) | 0.09 (0.03) | 0.12 (0.11) | **F = 1.35; df =35; p = 0.25** |
| Grey matter | 0.57 (0.08) | 0.60 (0.12) | 0.65 (0.14) | 0.61 (0.09) | **F = 2.72; df = 35; p = 0.11** |
| Cerebrospinal fluid | 0.30 (0.12) | 0.29 (0.12) | 0.26 (0.13) | 0.26 (0.11) | **F = 0.02; df = 35; p = 0.90** |
| Cramér Rao Lower Bounds (%) | | | | | |
|  | **Patients** | | **Healthy volunteers** | |  |
|  | **Pre** | **Post** | **Pre** | **Post** | **Group x Time Interaction** |
| Glutamate + glutamine | 6.50 (1.25) | 7.39 (1.46) | 7.13 (2.26) | 7.00 1.89) | **F = 1.73; df = 35; p = 0.09** |
| Glutamate | 6.58 (1.26) | 6.74 (1.33) | 6.60 (2.06) | 6.10 (0.90) | **F = 1.47; df = 35; p = 0.23** |
| N-acetyl aspartate | 3.16 (0.83) | 3.05 (0.41) | 2.94 (0.64) | 3.11 (1.02) | **F = 0.74; df = 35; p = 0.40** |

**eTable 3** Signal-to-noise ratio, Cramér Rao Lower Bounds, and voxel white matter, grey matter, and cerebrospinal fluid fractions in the anterior cingulate cortex in patients and controls pre- and post- riluzole. Data are presented as mean (standard deviation) and the statistical analysis shows the results of the group by time interaction tested using a repeated measures ANOVA.

|  | Pre-riluzole | Post-riluzole | Dependent t test |
| --- | --- | --- | --- |
| Patients | 22.69 (7.05) | 19.52 (4.79) | **t = 2.08; df = 35; p = 0.05** |
| Healthy volunteers | 20.37 (4.19) | 21.57 (4.97) | **t = 0.86; df = 35; p = 0.40** |
| Independent t test | **t = 1.21; df = 35; p = 0.24** | **t = -1.27; df = 35; p = 0.21** |  |

**eTable 4:** Post-hoc pre- vs post-riluzole comparison of anterior cingulate cortex Glutamate + glutamine (Glx) levels in patients and controls. Data are presented as mean, standard deviation, and independent/dependent t tests.

|  | Patients | | Healthy Volunteers | |  |
| --- | --- | --- | --- | --- | --- |
|  | Pre | Post | Pre | Post | **Group x Time Interaction** |
| Glutamate | 15.91 (4.10) | 14.89 (2.56) | 15.21 (2.93) | 16.00 (2.90) | **F = 2.13; df = 35; p = 0.15** |
| N-acetyl aspartate | 15.52 (3.30) | 14.70 (2.48) | 14.84 (3.32) | 14.81 (2.32) | **F = 0.55; df = 35; p = 0.46** |

**eTable 5** Cerebrospinal fluid corrected metabolite values in the anterior cingulate cortex pre- and post-riluzole. Data are presented as mean (standard deviation) and the statistical analysis shows the results of the group by time interaction tested using a repeated measures ANOVA.

|  | Patients | | Healthy volunteers | |  |
| --- | --- | --- | --- | --- | --- |
|  | **Pre** | **Post** | **Pre** | **Post** | **Group x Time Interaction** |
| Glutamate + glutamine/Cr | 1.76 (0.27) | 1.66 (0.29) | 1.77 (0.20) | 1.86 (0.31) | **F = 3.27; df = 35; p = 0.08** |
| Glutamate/Cr | 1.25 (0.18) | 1.28 (0.13) | 1.33 (0.21) | 1.39 (0.16) | **F = 0.12; df =35; p = 0.74** |
| N-acetyl aspartate/Cr | 1.22 (0.13) | 1.26 (0.10) | 1.28 (0.10) | 1.29 (0.09) | **F = 0.33; df =35; p = 0.57** |

**eTable 6** Creatine scaled metabolite values in the anterior cingulate cortex (ACC) before and after riluzole. Data are presented as mean (standard deviation) and the statistical analysis shows the results of the group by time interaction tested using a repeated measures ANOVA.

|  | Patients | Healthy volunteers | Independent t test |
| --- | --- | --- | --- |
| Pre-riluzole | 40.17 (6.67) | 39.48 (5.81) | **t = 0.34; df = 35; p = 0.74** |
| Post-riluzole | 40.85 (7.22) | 39.52 (5.25) | **t = 0.64; df = 35; p = 0.53** |
| Dependent t test | **t = 0.53; df = 35 p = 0.60** | **t = 0.03; df = 35 p = 0.97** |  |

**eTable 7:** Post-hoc examination of effect of riluzole on whole brain cerebral blood flow. Data are presented as mean, standard deviation, and independent/dependent t tests.

|  | Patients | Healthy volunteers | Independent t test |
| --- | --- | --- | --- |
| Pre-riluzole | 44.99 (7.51) | 44.03 (6.36) | **t = -0.42; df = 35; p = 0.68** |
| Post-riluzole | 45.13 (7.34) | 43.93 (5.02) | **t = -0.58; df = 35; p = 0.57** |
| Dependent t test | **t = 0.11; df =35 p = 0.91** | **t = 0.07; df =35 p = 0.94** |  |

**eTable 8:** Post-hoc examination of effect of riluzole on anterior cingulate cortex cerebral blood flow. Data are presented as mean, standard deviation, and independent/dependent t tests.

| Treatment Resistant Schizophrenia | Spearman’s correlation coefficient |
| --- | --- |
| Glx and PANSS +ve | r = -0.29; p =0.21 |
| Glx and PANSS -ve | r = 0.49; p = 0.03 |
| Glx and PANSS gen | r = -0.08; p = 0.75 |
| Glx and PANSS total | r = 0.08; p = 0.74 |
| Glx and AVLT total score | r = -0.63; p = 0.002 |
| ACC-BA10 connectivity and PANSS +ve | r = 0.22; p =0.37 |
| ACC-BA10 connectivity and PANSS -ve | r = -0.32; p = 0.17 |
| ACC-BA10 connectivity and PANSS gen | r = 0.07; p = 0.77 |
| ACC-BA10 connectivity and PANSS total | r = 0.05; p = 0.82 |
| ACC-BA10 connectivity and AVLT total score | r = 0.47; p = 0.04 |
| Healthy Volunteers |  |
| Glx and AVLT total score | r = -0.24; p = 0.41 |
| ACC-BA10 connectivity and AVLT total score | r = 0.28; p = 0.33 |

**eTable 9:** Relationship between baseline (pre-riluzole) ACC-Glx levels and clinical/cognitive rating scales, and ACC-BA10 functional connectivity and clinical/cognitive rating scales in patients. Relationship between ACC-Glx and ACC-BA10 connectivity with AVLT total scores in the healthy volunteer group alone is also presented.

**
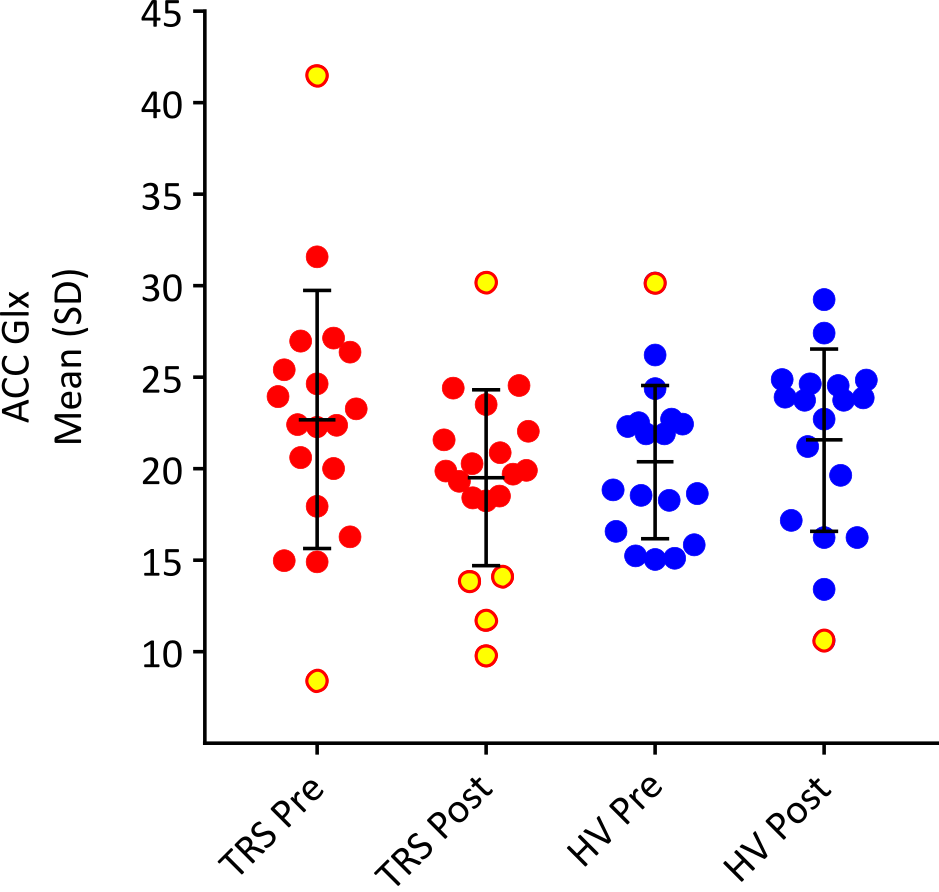
**

**eFigure 1** Anterior cingulate cortex CSF corrected glutamate + glutamine (Glx) in patients and healthy volunteers, before and after riluzole challenge. Outliers are depicted as yellow symbols, identified using the Tukey method.^3^

**References**

1. Howes OD, McCutcheon R, Agid O, de Bartolomeis A, van Beveren NJM, Birnbaum ML *et al.* Treatment-Resistant Schizophrenia: Treatment Response and Resistance in Psychosis (TRRIP) Working Group Consensus Guidelines on Diagnosis and Terminology. *Am J Psychiat* 2017; **174**(3)**:** 216-229.

2. Margulies DS, Kelly AM, Uddin LQ, Biswal BB, Castellanos FX, Milham MP. Mapping the functional connectivity of anterior cingulate cortex. *Neuroimage* 2007; **37**(2)**:** 579-588.

3. Tukey J. Exploratory data analysis. *Addison-Wesely* 1977.
